# The effect of sodium valproate on the biochemical parameters of reproductive function in male albino Wistar rats

**DOI:** 10.4103/0253-7613.45149

**Published:** 2008

**Authors:** P. Vijay, R. Yeshwanth, K.L. Bairy

**Affiliations:** Department of Anatomy, Maleka Manipal Medical College, Manipal, India; 1 Department of Pharmacology, Maleka Manipal Medical College, Manipal, India; 2 Department of Pharmacology, Kasturba Medical College, Manipal, India

**Keywords:** Lactate dehydrogenase, sodium valproate, testosterone

## Abstract

**Objective:**

To assess the effects of sodium valproate on intratesticular testosterone and lactic dehydrogenase level in rats.

**Methods:**

Male Wistar rats (12 weeks old) were treated with sodium valproate and sacrificed at the end of the 2^nd^, 4^th^, 5^th^, 7^th^, 10^th^ and 15^th^ week, after the last exposure to sodium valproate. The testes were removed, weighed and processed for biochemical analysis.

**Results:**

The intratesticular testosterone level was significantly (*P*<0.001) reduced in 200 mg/kg and 400 mg/kg treated rats. The intratesticular lactate dehydrogenase (LDH) level was significantly (*P*<0.001) increased by valproate in a time dependent manner.

**Conclusion:**

Valproate causes reversible change in intratesticular testosterone and LDH level.

## Introduction

Clinically, the most prominent effects of anticonvulsant medications on endocrine function are those on sexuality and the reproductive function. The hepatic enzyme-inducing antiepileptic drugs (AEDs) - phenobarbital, primidone, phenytoin, and carbamazepine - may all contribute to or cause reproductive and sexual dysfunction in men with epilepsy.[1,2] Valproate, an enzyme inhibitor, has also been associated with altered androgen levels,[3] as well as altered reproductive parameters.[4,5] The maintenance of adult mammalian spermatogenesis is dependent upon the steroid hormone testosterone, which is produced by testicular leydig cells, in response to the secretion of pituitary luteinizing hormone.[6] Previous studies of spermatogenesis in rats have shown that the experimental reduction of intratesticular testosterone to low enough levels results in germ cell loss[7–9] and that the readministration of testosterone restores spermatogenesis.[10,11] Intratesticular testosterone is thought to play a very important role in spermatogenesis; however, it is very rarely measured in patients receiving AEDs.

Lactate dehydrogenase (LDH) is a ubiquitous enzyme present in both plants and animals. Eliasson and Virgi[12] suggested that quantitative analysis of the isoenzyme lactate dehydrogenase - C4 in semen from fertile and infertile men may provide a guide regarding the status of the seminiferous epithelium and the degree of germ cell degeneration. The estimation of LDH levels provides a quantitative basis for the loss of cell viability and its application in assessing the cytotoxicity of the cell.[13,14] Most of the studies on sodium valproate indicate that it is gonadotoxic and hence can affect fertility. However, all these reports are at one point sampling and there are no reports regarding how long the effects last after exposure to the drug. Hence, a study was planned to assess the effects of sodium valproate at different sampling time on the biochemical markers of testicular function in male Wistar rats.

## Materials and Methods

### Animals

Twelve-week-old male Wistar rats (150-200g), bred locally in the central animal house, were selected for the study. They were housed in propylene cages and were provided bedding with paddy husk. Temperature was maintained at 25 ± 1°C, with a humidity of 45 ±1%. The animals had free access to sterile food (animal chow) and water *ad libitum.* Animal care and handling was done as per the guidelines set by the Indian National Academy, New Delhi, India. The study was started after getting clearance from the Institutional Animal Ethics Committee.

A total of 108 rats were segregated to 18 groups of 6 animals each. Six groups each were treated with 0.1 ml of distilled water, sodium valproate 200 mg and sodium valproate 400mg for 60 days (n = 6/group/dose/sample time). The powdered form of sodium valproate was obtained from Knoll Pharmaceuticals Ltd, Mumbai. The sodium valproate was dissolved in distilled water and administered orally. The median lethal dose of sodium valproate in rodents varies between 1100 and 3900 mg/kg body weight.[15] The dose and route of administration was based on earlier reports.[16,17] The rats were sacrificed by terminal anesthesia (pentobarbital sodium, 45mg/kg) at the end of the 2nd, 4^th^, 5^th^, 7th, 10th and 15^th^ week, after the last exposure to sodium valproate.

### Preparation of tissue homogenate

Testes were removed and weighed. The testes were then minced in phosphate buffer solution at a ratio of 1: 10, using pestle and mortar. The tissue homogenate obtained was cold centrifuged. The supernatant was taken for the estimation of intratesticular testosterone and intratesticular lactate dehydrogenase.

### Estimation of intratesticular testosterone level

The testicular level of testosterone was analyzed in the homogenate by using a kit designed for ELISA (ElAgen Testosterone-Biochem Immunosystem, Italia S.P.A.). About 50μl of calibrators and tissue homogenate sample were added to appropriate wells of strips. About 200μl of horseradish peroxidase - testosterone conjugate was added to each well in sequence. The mixture was incubated for two hours at 37°C, without covering the plate. Following this, the solution was discarded, the wells were rinsed thrice with washing solution (Tween 20) and amphotericin-B (2.5μg/ml) in citrate- borate buffer) and the residual fluid was removed. Immediately, 100 μl of chromogen substrate mixture (0.26mg/ml of 3, 3', 5, 5'- tetramethyl benzidine and 0.01% (w/v) of hydrogen peroxide in citrate buffer) was added to the wells and incubated for 15 min at room temperature, avoiding exposure to sunlight. Reaction was stopped by pipetting 100 μl of the stop solution (sulfuric acid-0.3mol/l) into the wells. Absorption was read in ELISA at 450 nm, within one hour from the addition of stop solution, as per the manufacturer's instructions.

### Estimation of intratesticular lactate dehydrogenase level

Testicular lactate dehydrogenase (LDH) was estimated by Optimized standard kit method (Roche/Hitachi), based on the principle that lactate dehydrogenase catalyses the conversion of pyruvate to lactate; reduced Nicotinamide Adenine dinucleotide (NADH) is oxidized to Nicotinamide Adenine dinucleotide (NAD) in the process. The rate of decrease in NADH is directly proportional to the LDH activity. The LDH activity was estimated by a kit using a spectrophotometer (Optimized Standard Kit; Roche/Hitachi).

### Statistical analysis

For each group, six animals were used and mean ± SD (standard deviation) was calculated. Results obtained from the present study were correlated and analyzed by One Way Analysis of Variance (ANOVA). Values of *P*< 0.05 were considered statistically significant.

## Results

**Figure 1:**
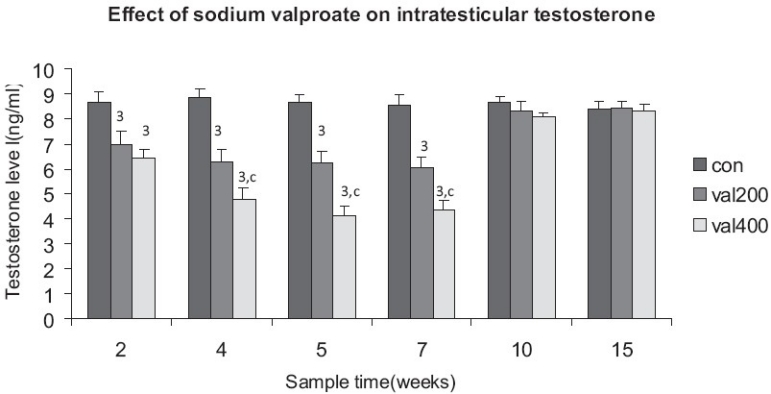
Time response relationship for sodium valproate induced changes in testosterone level. Each time at particular dose represents mean +SD from six animals. Significant values are: normal control vs. treated, 3 = *P*<0.001; 200mg vs. 400mg, c = *P*<0.001

The intratesticular testosterone was significantly reduced in rats treated with 200mg/kg and in rats treated with the 400mg/kg. Significant difference was also seen in the levels of intratesticular testosterone between the treated groups. Significant difference between the treated groups was observed till the 7^th^ week. Recovery period for both the doses took a longer time and reached normal values only by the 15^th^ week [Figure 1]. The decline in intratesticular testosterone level was the highest during the 5^th^ and the 7^th^ week, for both the dose levels.

**Figure 2:**
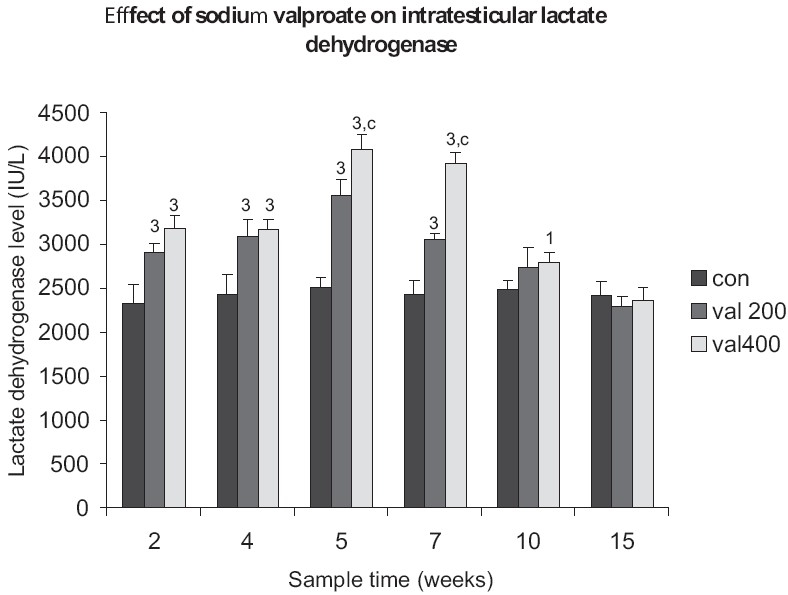
Time response relationship for sodium valproate induced changes in lactate dehydrogenase level. Each time at particular dose represents mean +SD from six animals. Significant values are: normal control vs. treated, 1= *P*< 0.05, 3 = *P*<0.001; 200mg vs. 400mg, c = *P*<0.001

The intratesticular LDH level was significantly increased by valproate, in a dose dependent manner in the 2^nd^ week. A similar observation was made in the following sampling weeks and complete recovery to normal values was reached only by the 15^th^ week. The elevation of LDH level was the highest in the 5^th^ and 7^th^ week. Significant differences between the groups were observed during the 5^th^ and 7^th^ week [Figure 2].

## Discussion

Data generated clearly shows that the levels of intratesticular testosterone in rats treated with sodium valproate decreased significantly in the 2^nd^ to 7^th^ week sampling time. Bauer *et al*.[4] and Kuhn-Velten *et al*.[18] reported that valproate acts directly on the testis, to inhibit testosterone synthesis by the leydig cells. In the present study, it is more than likely that a similar effect has been responsible for the low intratesticular testosterone level. The testosterone-to-LH ratio is a sensitive measure of testicular function. It is low in men with temporal lobe epilepsy not taking AEDs, but is even more abnormal in this same population with the use of valproate.[4] However, the latter possibility can be ruled out, since the present study was carried out on nonepileptic rats.

Although incompletely researched, it has been suggested that AEDs induce the production of the enzyme aromatase in the liver. This enzyme converts testosterone to estradiol (the final common path of all natural estradiol production). Induction of aromatase production leads to an elevated serum level of estradiol.[19] By shunting free testosterone to estradiol, serum free testosterone level is further reduced. Thus, the ratio of free testosterone to estradiol is lower in men with epilepsy and hyposexuality than in sexually normal epilepsy patients or in normal controls.[20] Estradiol may impair testosterone secretion by suppressing male luteinizing hormone secretion or by producing premature aging of the hypothalamic arcuate nucleus. However, it is not known whether the antiepileptic drugs can cause the conversion of testosterone to estradiol within the testes or whether it is a process only in the serum. So the decrease in the levels of intratesticular testosterone observed in the present study may be mainly due to the direct effect of these drugs on the leydig cells and, at the same time, the possible conversion of intratesticular testosterone to estrogen by the enzyme aromatase cannot be ignored.

Lactate dehydrogenase (LDH) enzyme is widely distributed throughout the body; cellular damage causes an elevation of the total serum LDH. When disease or injury affects tissues containing LDH, the cells release LDH into the bloodstream, where it is identified in higher than normal levels. Eliasson and Virgi[12] suggested that quantitative analysis of the isoenzyme lactate dehydrogenase -C4 in semen from fertile and infertile men may provide a guide regarding the status of the seminiferous epithelium and the degree of germ cell degeneration. LDH - C4 is a testes specific enzyme; however, in the present study, the estimation was done on the total LDH.

Goddard *et al*.[21] showed that adult rats treated with flutamide (an antiandrogen) in utero induced altered spermatogenesis. However, the levels of LDH were decreased, which is contrary to the earlier reports. They further suggest that this decrease in LDH indicates that transport of lactate produced by sertoli cells to the germ cells could be altered. In the present study, it was observed that the LDH level was increased in a significant manner during the 2^nd^ to 7^th^ week sampling time. When the germ cells which possess LDH - C4 degenerates, some of the enzymes leak into the seminiferous tubule fluid and eventually find their way into the semen. The estimation of LDH levels provides a quantitative basis for the loss of cell viability and its application in assessing the cytotoxicity of the cell.[13,14] According to Sinha *et al*. and Pant and Srivastava,[22,23] the increase in LDH activity level has a direct effect on testicular functions such as sperm count and sperm production, as well as sperm morphology. This indicates the cytotoxicity of sodium valproate.

The present study concludes that even though valproate decreases the fertility by affecting the germ cells, somatic cells, intratesticular testosterone and LDH, these effects are not permanent and are reversed once the drug is withdrawn. This finding has a clinical relevance because of the widespread use of valproate in the management of epilepsies and also its use in bipolar disorders. Future studies should focus on the effect of this drug on other hormones and enzymes, as well as its effect on other reproductive parameters.

## References

[1] Toone BK, Wheeler M, Nanjee M, Fenwick P, Grant R (1983). Sex hormones, sexual activity and plasma anticonvulsant levels in male epileptics. J Neurol Neurosurg Psychiatry.

[2] Isojarvi JI, Pakarinen AJ, Ylipalosaari PJ, Myllylä VV (1990). Serum hormones in male epileptic patients receiving anticonvulsant medication. Arch Neurol.

[3] Rattya J, Turkka J, Pakarinen AJ, Knip M, Kotila MA, Lukkarinen O (2001). Reproductive endocrine effects of valproate, carbamazepine, and oxcarbazepine in men with epilepsy. Neurology.

[4] Bauer J, Blumenthal S, Reuber M, Stoffel-Wagner B (2004). Epilepsy syndrome, focus location, and treatment choice affect testicular function in men with epilepsy. Neurology.

[5] Isojarvi JI, Lofgren E, Juntunen KS, Pakarinen AJ, Päivänsalo M, Rautakorpi I (2004). Effect of epilepsy and antiepileptic drugs on male reproductive health. Neurology.

[6] Zirkin BR, Santulli R, Awoniyi CA, Ewing LL (1989). Maintenance of advanced spermatogenic cells in the adult-rat testis—quantitative relationship to testosterone concentration within the testis. Endocrinology.

[7] Tapanainen JS, Tilly JL, Vihko KK, Hsueh AJ (1989). Hormonal control of apoptotic cell death in the testis: gonadotropins and androgens as testicular cell survival factors. Mol Endocrinol.

[8] Henriksen K, Hakovirta H, Parvinen M (1995). Testosterone inhibits and induces apoptosis in rat seminiferous tubules in a stage-specific manner: In situ quantification in squash preparations after administration of ethane dimethane sulfonate. Endocrinology.

[9] Sinha Hikim AP, Swerdloff RS (1999). Hormonal and genetic control of germ cell apoptosis in the testis. Rev Reprod.

[10] Awoniyi CA, Santulli R, Sprando RL, Ewing LL, Zirkin BR (1989). Restoration of advanced spermatogenic cells in the experimentally regressed rat testis: Quantitative relationship to testosterone concentration within the testis. Endocrinology.

[11] Awoniyi CA, Sprando RL, Santulli R, Chandrashekar V, Ewing LL, Zirkin BR (1990). Restoration of spermatogenesis by exogenously administered testosterone in rats made azoospermic by hypophysectomy or withdrawal of luteinizing hormone alone. Endocrinology.

[12] Eliasson R, Virji N (1985). LDH- C4 in human seminal plasma and its relationship t testicular function, II: Clinical aspects. Int J Androl.

[13] Decker T, Lohmann-Matthes ML (1988). A quick and simple method for the quantitation of lactate dehydrogenase release in measurements of cellular cytotoxicity and tumor necrosis factor (TNF) acticity. J Immunol Methods.

[14] Adiga SK, Jagetia GC (1999). Effect of teniposide (Vm-26) on the cell survival, micronuclei induction and lactate dehydrogenase activity on V79 cells. Toxicology.

[15] Walker RM, Smith GS, Barsoum NJ, Macallum GE (1990). Preclinical toxicology of the anticonvulsant calcium valproate. Toxicology.

[16] Sveberg Roste L, Tauboll E, Berner A, Berg KA, Aleksandersen M, Gjerstad L (2001). Morphological changes in the testis after long-term valproate treatment in male Wistar rats. Seizure.

[17] Sveberg Roste L, Tauboll E, Isojarvi JI, Pakarinen AJ, Huhtaniemi IT, Knip M (2002). Effects of chronic valproate treatment on reproductive endocrine hormones in female and male Wistar rats. Reprod Toxicol.

[18] Kuhn-Velten WN, Herzog AG, Muller MR (1990). Acute effects of anticonvulsant drugs on gonadotropin-stimulated and precursor-supported testicular androgen production. Eur J Pharmacol.

[19] Herzog AG, Levesque L, Drislane F, Ronthal M, Schomer DL (1991). Phenytoin-induced elevations of serum estradiol and reproductive dysfunction in men with epilepsy. Epilepsia.

[20] Murialdo G, Galimberti CA, Fonzi S, Manni R, Costelli P, Parodi C (1995). Sex hormones and pituitary function in male epileptic patients with altered or normal sexuality. Epilepsia.

[21] Goddard I, Florin A, Mauduit C, Tabone E, Contard P, Bars R (2003). Alteration of lactate production and transport in the adult rat testis exposedin utero to flutamide. Mol Cell Endocrinol.

[22] Sinha H, Narayana R, Shanker R, Saxena DK (1995). Endosulfan induced biochemical change in the testes of rats. Vet Hum Toxicol.

[23] Pant N, Srivastava SP (2003). Testicular and spermatotoxic effects of quinalphos in rats. J Appl Toxicol.

